# Lectures on the Diagnosis and Treatment of Diseases of the Heart

**Published:** 1841-10-30

**Authors:** W. W. Gerhard


### MEDICAL EXAMINER.

DEVOTED TO MEDICINE, SURGERY, AND THE COLLATERAL SCIENCES.

No. 44.] PHILADELPHIA, SATURDAY, OCTOBER 30, 1841. [Vol. IV.

### LECTURES ON THE DIAGNOSIS AND TREATMENT OF DISEASES OF THE HEART.

BY W. W. GERHARD, M. D.

### LECTURE XVII.

#### EXAMINATION OF THE HEART.

The heart requires to be examined under several different points of view. The most important of these are its position in the thorax, and the relative situation of the parts of it; the size of the heart, as ascertained by percussion; the impulsion; the sounds, and their rhythm or succession; lastly, the mode in which the heart acts, whether in a regular or in a spasmodic, ill-defined manner. Besides these signs, which are strictly physical, we learn much as to the diseases of the heart from the sensations complained of at the præcordial region, the respiration and the impediments to it, the capillary and venous circulation, and, lastly, the surrounding disorders of the whole body, which arise from the cardiac region.

1. *Position of the Heart.*—In general terms, it may be stated that the heart is situated at the lower portion of the sternum, including nearly the whole breadth of this bone, and extending to the left of it, a short distance within the nipple; in height, the heart reaches from the intercostal space between the third and fourth ribs, to the base of the thorax. It occupies, therefore, the anterior and lower portion of the left side of the thorax, in a definite extent, and this portion so occupied is called the præcordial region, or, in other words, the region of the heart. The exact situation of the heart, and the relative position of its different parts and valves, are readily seen by reference to the annexed plate.

2. *Size of the Heart.*—The size of the heart is known by two phenomena, the anormal prominence formed at the cartilages of the fifth, sixth, and seventh ribs, and the dulness on percussion, which may become greater or less than it should be in the healthy state of an individual of a given fever and embonpoint. By the size of the heart we understand the whole taken collectively; hence, an enlargement of the muscular substance does not differ from the more external distension caused by effusions of pus or serum into the pericardium. Then the increase in dimensions is very considerable, the chest is thrust forwards, but the form of the prominence is different; when there is an effusion into the pericardium it is pyramidal, the apex of the pyramid at the upper portion; when it depends upon a real increase of the muscular structure, the projection is in general less marked, but more diffused, and forms an oval, the long diameter of which extends laterally, instead of vertically, as in the former case. The more exact mode of estimating enlargement of the heart is, however, by percussion. This is readily enough practiced, but to render it of practical benefit, the observer should retain an accurate recollection of the average normal dimensions in an individual of the general health and condition of the patient. It is true that the general health may have a very decided effect upon the heart, as well as upon the rest of the organs, but it here suffers with the rest, and the change is limited to a slight augmentation or diminution, corresponding with that of the muscular structure in general. The real advantages of percussion are much more decided when we desire to learn the condition of the heart, and its state as an individual organ. To do this, we must recollect that in the healthy individual the heart is not entirely overlapped by the lungs, but that a portion of its structure, as is seen by the plate, which is chiefly composed of the anterior surface of the right ventricle, is in immediate contact with the parietes of the chest. This portion varies in extent, partly from changes in the condition of the heart and pericardium, partly from lesions of the lungs and pleura. Thus, without any actual change in the heart, the percussion at the præcordial region may be rendered very dull from induration of the lungs, or pleuritic effusion, while emphysema may enlarge the lungs, and render the sound preternaturally clear in the region of the heart. A little attention to the condition of the lungs will in general obviate the chances of error.

The heart itself, when diseased, often produces a decided change in the results of percussion, which then depend upon alterations of form. In the natural state, the extent of dulness does not exceed a space of about two inches in length, measured along the sternum, and an inch and a half laterally; that is, the dulness extends to a short distance within the nipple, and about the middle of this space, just at the right margin of the sternum, it amounts in most persons almost to flatness. If the heart be enlarged, or if an effusion of liquid has taken place into the pericardium, the dulness is of course increased in proportion to the degree of enlargement or increase. When the dulness results from hypertrophy of the heart, it is more rounded in its shape than when it depends upon pericardial effusion; in the latter case, the original shape of the serous sac is still preserved, and the space in which the dulness is most evident is pyramidal, the apex of the pyramid being towards the upper part of the chest. The mathematical exactness of this mode of measuration is readily ascertained by a process which was very carefully pursued by Dr. Pennock a few years since; that is, forcing long needles at the limits of the dulness, and examining the parts perforated by them after the body was opened.

*Impulsion.*—The impulsion of the heart furnishes one of the least complicated sets of signs connected with this organ. It is produced, as is well known, by the contraction or systole of the heart, during which the point of the organ is quickly impelled against the ribs, striking near the cartilages of the fifth and sixth ribs a little within the nipple. In the healthy state of the heart, the blow or impulse is given almost exclusively by the point of the heart; hence the sensation is sharp and decided, as would naturally be caused by the quick stroke of a small surface against the thoracic parietes. If the heart is thrown into violent action by quick exercise, or by nervous irritability, the impulsion is increased in force, but the surface upon which it falls is still very limited in extent; should the bulk of the heart and the quantity of muscular tissue be increased, as in cases of hypertrophy, the momentum of the impulsion is much increased, but it is diffused over a much larger surface than in the normal condition of the organ, and the mass of the heart is applied slowly against the chest, as it were point after point, so as to give it a heaving or waving motion, instead of a sharp, clear impulsion. In low fevers, and in other diseases in which the powers of life and the strength of the patient are much diminished, the impulsion of the heart is decidedly lessened, and its force nearly destroyed. The diminished impulsion is then a good oracle in therapeutics, and affords us one of the first indications for a supporting treatment. The impulsion of the heart, it is evident, can only serve as a diagnostic sign, when the patient is still possessed of a moderate degree of strength; hence, in cases in which the muscular substance is really much increased, the force of the blow may be so much diminished that no stronger impulsion is made upon the parietes of the thorax than would result from a heart, which is not at all enlarged.

